# Two Years of Active Pharmacovigilance Surveillance and Therapeutic Reconciliation in Frail Populations: The MEAP 3.0 Study

**DOI:** 10.3390/jcm12237447

**Published:** 2023-11-30

**Authors:** Anna Bombelli, Greta Guarnieri, Niccolò Lombardi, Maria Giuseppa Sullo, Edoardo Spina, Giada Crescioli, Concetta Rafaniello, Giuseppe Cicala, Veronica Marangon, Rachele Folchino, Silvia Vecchio, Giulia Mosini, Sonia Radice, Emilio Clementi, MEAP 3.0 Group

**Affiliations:** 1 Pharmacovigilance & Clinical Research, International Centre for Pesticides and Health Risk Prevention, ASST Fatebenefratelli-Sacco, Department of Biomedical and Clinical Sciences, Università degli Studi di Milano, 20157 Milan, Italy; annabombelli8@gmail.com (A.B.); greta.guarnieri@unimi.it (G.G.); sonia.radice@unimi.it (S.R.); emilio.clementi@unimi.it (E.C.); 2 Pharmacovigilance and Pharmacoepidemiology Research Unit, Section of Pharmacology and Toxicology, Department of Neurosciences, Psychology, Drug Research and Child Health, University of Florence, 50139 Florence, Italy; niccolo.lombardi@unifi.it (N.L.); giada.crescioli@unifi.it (G.C.); 3 AOU Policlinico, Università degli Studi della Campania “L. Vanvitelli”, 80138 Naples, Italy; mariagiuseppa.sullo@unicampania.it (M.G.S.); concetta.rafaniello@unicampania.it (C.R.); 4 Department of Clinical and Experimental Medicine, University of Messina, 98125 Messina, Italy; edoardo.spina@unime.it (E.S.); giuseppe.cicala@unime.it (G.C.); 5 Pharmacovigilance Unit, Agency for Health Protection (ATS) Monza e Brianza, 23900 Lecco, Italy; veronica.marangon@ats-brianza.it; 6 Pharmacy Department, Agency for Health Protection (ATS) Milan, 20161 Milan, Italy; rfolchino@ats-milano.it; 7 Unit of Control of Territorial Pharmaceutical and Prosthetic Performances-HTA, Health Protection Agency (ATS) Pavia, 27100 Pavia, Italy; silvia_vecchio@ats-pavia.it; 8 Scientific Institute, IRCCS E. Medea, 23842 Bosisio Parini, Italy

**Keywords:** adverse drug reaction, drug–drug interaction, paediatric, elderly, frail populations, pharmacovigilance, polypharmacy, therapeutic reconciliation, personalised medicine

## Abstract

Awareness related to the risk/benefit profile of therapies used in paediatric and elderly patients is limited. We carried out a study, called the MEAP 3.0 study, to collect and analyse evidence of adverse drug reactions (ADRs) and drug–drug interactions (DDIs) that occurred in frail populations under polypharmacy in a real-world setting. Data were retrieved from reports of ADRs and pharmacological counselling from patients treated in hospitals and territorial health services. We collected 2977 ADRs reports and identified ‘anti-infectives for systemic use’ and ‘cardiovascular system’ as the most frequently implicated pharmacological classes in under-18 and over-65 patients, respectively. We detected 2179 DDIs, of which 10.7% were related to at least one ADR: 22 were classified as ‘contraindicated’ (7 in the paediatric group and 15 in the elderly one), and 61 as ‘major’ (6 in the paediatric patients and 55 in the geriatric ones), while 151 DDIs were classified as ‘moderate’ (10 referred to paediatric population, and 109 to elderly patient) and as ‘minor’ (1 in paediatric patients, and 31 in the elderly ones). The MEAP 3.0 project demonstrates that pharmacovigilance surveillance and therapeutic reconciliation are valid strategies to avoid potential DDIs and the occurrence of ADRs, allowing for personalised medicine.

## 1. Introduction

Drug consumption has been increasing globally, and it is set to rise worldwide as a result of the ageing of the population [1]. Ageing is associated with an increased prevalence of multiple chronic diseases for which multiple treatments need to be prescribed [1,2,3]. Up to 30–60% of elderly patients, i.e., those over 65 years of age, are currently treated with five or more medicines [4]. Another critical issue related to the therapy management of elderly patients is the possible similarity of disease symptoms with those induced by the polytherapy. Discrimination between drug-induced events and symptoms during the course of the disease is critical as failure to do so could lead to a kind of inappropriate prescribing, known as ‘prescribing cascade’: this consists of a sequence of events among which an adverse drug event is misinterpreted as a new medical condition, leading to the addition of another, potentially avoidable, medication [5,6].

Polypharmacy is also highly prevalent among paediatric patients, especially hospitalised ones. Moreover, hospitalised children could be also characterised by physiological dysfunctions due to chronic conditions, the management of which is complex.

A further level of complexity in understanding and evaluating drug-induced events is generated by the known physiological and hence pharmacokinetic diversity of the two populations with respect to the middle-aged adult. Elderly people are generally characterised by alterations in all phases of pharmacokinetic processes, mainly due to the reduction in several homeostatic mechanisms and the loss of renal and hepatic function [7]. Likewise, ageing is associated with a reduction in the number of receptors, resulting in signal transduction and drug response alterations [8]. Childhood is also associated with distribution and absorption alterations which differ depending on the paediatric age range [9]. Furthermore, it is particularly difficult to predict pharmacological effects in children as development occurs quickly, bringing about rapid changes in drug metabolism [1,10]. These physiological changes could be associated with a potentially reduced drug responsiveness and an increased risk of drug-induced events.

Given the above reasons, age-related changes, together with comorbidities and polypharmacy, make paediatric and elderly patients more susceptible to unexpected adverse drug reactions (ADRs) and potential drug–drug interactions (DDIs).

The Pharmacovigilance Service of the L. Sacco University Hospital, based on its previous experience [11] and in collaboration with the Italian Medicines Agency (AIFA) and the Pharmacovigilance Regional Centres of Lombardy, Campania, Tuscany, and Sicily developed a nationwide pharmacovigilance project, named ‘Monitoring of adverse events on the frail populations’ (MEAP 3.0) [11].

Spontaneous reporting is an essential tool to address the lack of evidence in elderly and paediatric populations drugs’ safety profile: elderly patients with comorbidities and paediatric patients are generally excluded from clinical trials and the resulting evidence may not be generalised to these populations [9,12], leading to an empirical approach to the therapeutic management. Although paediatric regulation has increased the number of clinical trials in children, off-label use is still widely common in paediatric settings [13], leading to an increased risk of unexpected ADRs occurrence.

MEAP 3.0 was therefore designed to increase information based on real-world evidence to investigate potential DDIs and to better characterise patterns of related ADRs in these heterogeneous and critical populations. Furthermore, it provided consultancy by pharmacologists on DDIs to the involved physicians. This pharmacovigilance study has generated a valuable source of real-world data that could be useful in gaining new insights on the safety profile in geriatric and paediatric patients, as well as in tailoring therapies through the pharmacological counselling tool (Supplementary Figure S1).

## 2. Materials and Methods

### 2.1. Data Source

MEAP 3.0 was a prospective active pharmacovigilance project to assess ADRs and DDIs in vulnerable populations, coordinated by the Pharmacovigilance Service of the University Hospital L. Sacco, in Italy.

It involved 6 Hospitals, 4 local Territorial Health Districts, and 2 scientific institutes of Campania, Lombardy, Tuscany, and Sicily, covering a significant fraction of the overall elderly and paediatric inpatients and outpatients of these regions. In each centre, physicians were informed about the aims of the MEAP 3.0 study, and a monitor selected by the pharmacovigilance centre was assigned to each structure. Bimonthly reports containing an overview of ADRs, patients’ features, and drug treatments were made available to all participating centres.

This study was performed on data retrieved by reports of suspected ADRs and pharmacological counselling related to individuals aged over 65 and under 18 years collected between 2020 and 2022.

Pharmacological counselling was introduced to support physicians in therapy decisions by means of an institutional form designed by the Pharmacovigilance Service of the L. Sacco University Hospital. The form was designed to contain patient personal information, ongoing therapy details (i.e., drug, dose, and frequency, route of administration, indication for use and duration), information related to the hospital unit of the applicant, and the clinical question. The clinical question could be related to the need to add a new drug in complex polytherapies, that could lead to a potential DDI, and/or to the investigation of the occurrence of a suspected ADR and/or to the information about the therapy administration, i.e., dose, frequency, and route of administration. The request could be sent by every hospital department via email or using the hospital management software to the Pharmacovigilance Service of the L. Sacco University Hospital which then examined the query posit in the form. Counselling was provided based on international DDIs checkers, summaries of product characteristics, and the scientific literature. The achieved results were then used to generate the MEAP 3.0 primary data source, an online database. It was set up at the beginning of the project with the purpose of collecting all identified reports and pharmacological counselling to ease data collection from each centre involved in the project.

### 2.2. Data Collection

The following information was collected by filling out multiple fields of the database:

- Reporting source;
- Patient information (i.e., date of birth, gender, concomitant diseases and comorbidities);
- Ongoing therapy (i.e., active principle, ATC, therapeutic indication, dose, route of administration, duration of therapy, therapeutic changes);
- Potential DDIs details (i.e., seriousness, type, mechanism, possible effects);
- In the case of the presence of potential DDIs, ADR-related information (i.e., seriousness and outcome).

DDIs seriousness was defined as ‘contraindicated’, ‘major’, ‘moderate’, or ‘minor’ based on the classification of INTERCheckWEB^®^ [14]. DDIs type could have pharmacodynamic (PD) or pharmacokinetic (PK) features. Regarding PD DDIs, the mechanism could be described as ‘additive effects/synergism’ or ‘indirect effect’ or ‘functional antagonism’ or ‘unknown’, while for PK DDIs, the mechanism could be described as ‘absorption induction/inhibition’ or ‘distribution alterations’ or ‘transport induction/inhibition’ or ‘CYP inhibition/induction’ or ‘phase 2 enzymes induction/inhibition’ or ‘clearance enhancement/reduction’ or ‘unknown’.

ADRs were classified as ‘serious’ or ‘non-serious’ according to the WHO Critical Term List [15]. Specifically, ADRs were considered ‘serious’ in the case of death, life-threatening, hospitalisation or prolonged hospitalisation, disability or permanent damage, congenital anomaly/birth defect, or another medically important condition. ADRs outcome was defined as ‘recovered/resolved’ or ‘recovering/resolving’ or ‘not recovered/not resolved’ or ‘recovered/resolved with sequelae’ or ‘fatal’ or ‘unknown’ or ‘not specified’.

ADRs, concomitant diseases, and DDIs possible effects were codified as detailed in the Medical Dictionary for Regulatory Activities (MedDRAs) and organised according to the System Organ Class (SOC) classification and Preferred Terms (PT). In particular, the analysis was based on the MedDRAs version 23.1 [16].

Each involved drug was classified according to the Anatomical Therapeutic Chemical (ATC) Classification System [17].

### 2.3. Data Analysis

Descriptive statistics were used to summarise the data. Continuous data were reported as mean values and standard deviation (SD), whereas categorical data were reported as frequencies and percentages. Patients were stratified into two groups: group 1 (paediatric patients: ≤18 years) and group 2 (elderly patients: ≥65 years).

## 3. Results

A total of 2977 ADRs reports was collected, of which 1513 (50.8%) were from paediatric patients and 1464 (49.2%) from elderly patients. The mean age of the paediatric patients was 6.8 years with a standard deviation of 5.8; no gender predominance was observed. The mean age in elderly patients was 75.7 years with a standard deviation of 7.6; a marked female predominance was observed (63.1%).

As shown in Table 1, the most frequently reported drugs in paediatric reports belonged to ‘anti-infectives for systemic use’ (88.6%) and ‘nervous system’ (22.3%) ATC groups. In elderly patients, the ATC groups with the highest percentage of reported drugs were ‘cardiovascular system’ (89.6%) and ‘anti-infectives for systemic use’ (68%).

Based on the MedDRAs SOCs, the main concomitant diseases in paediatric patients were ‘immune system disorders’, ‘nervous system disorders’, and ‘congenital, familial and genetic disorders’, while in elderly patients they were ‘vascular disorders’, ‘metabolism and nutrition disorders’, and ‘cardiac disorders’.

### 3.1. DDI

Of 2977 reports, 504 (16.9%) involved at least one interaction, of which 94 (18.7%) referred to paediatric patients and 410 (81.3%) to elderly people; 2179 potential DDIs were detected. In regard to the severity, 702 interactions (32.2%) were clinically relevant: 264 (12.1%) were classified as ‘contraindicated’ (70 in paediatric patients and 194 in elderly people) and 438 (20.1%) as ‘major’ (52 in paediatric patients and 386 in patients over 65 years old).

A total of 1147 (52.7%) DDIs were classified as ‘moderate’ (125 in paediatric reports and 1022 in elderly ones) and 330 (15.1%) as ‘minor’ (4 in paediatric patients and 326 in elderly patients).

Among the 2179 potential DDIs, 1588 (72.9%) were PD interactions, while 591 (27.1%) were PK DDIs.

In paediatric reports, ‘nervous system’ (27.3%), ‘alimentary tract and metabolism’ (21.3%), and ‘anti-infectives for systemic use’ (19.5%) were the most frequent ATC groups implicated in potential DDIs, and the possible consequences were related to the following MedDRAs SOCs: ‘cardiac disorders’ (33.2%), ‘investigations’ (24.9%), and ‘nervous system disorders’ (10.9%).

Among elderly patients, the most frequently implicated drugs in a potential DDI belonged to ‘cardiovascular system’ (36%), ‘blood and blood forming organs’ (17.2%), ‘alimentary tract and metabolism’ (17.1%), and ‘nervous system’ (15.5%) ATC groups, and the possible consequences were associated with the following MedDRAs SOCs: ‘cardiac disorders’ (24.2%), ‘investigations’ (21.5%), ‘metabolism and nutrition disorders’ (17.6%), and ‘vascular disorders’ (8.6%).

Regarding DDIs, 234 (10.7%) caused at least a related ADR (67.9% of ADRs were serious), and considering the relevant ones, 22 were classified as ‘contraindicated’ (7 in the paediatric group and 15 in the elderly one), and 61 as ‘major’ (6 in the paediatric patients and 55 in the geriatric ones).

In total, 151 DDIs related to ADRs were classified as ‘moderate’ (10 referred to the paediatric population and 109 to elderly patients) and as ‘minor’ (1 in paediatric patients and 31 in elderly ones).

**Figure 1:**
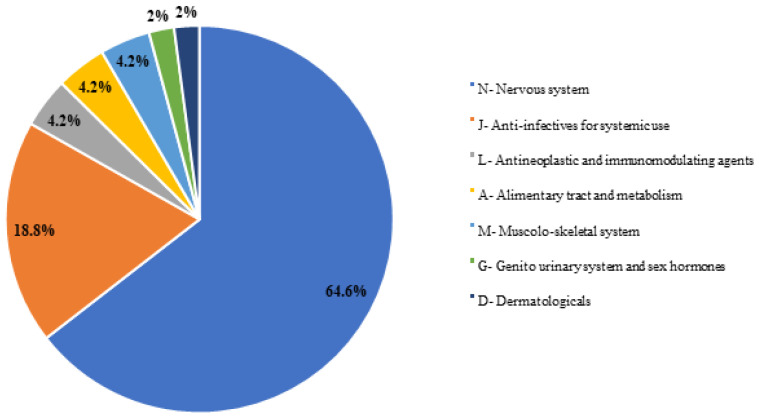
Drugs involved in DDIs in paediatric patients classified by ATC code.

**Figure 2:**
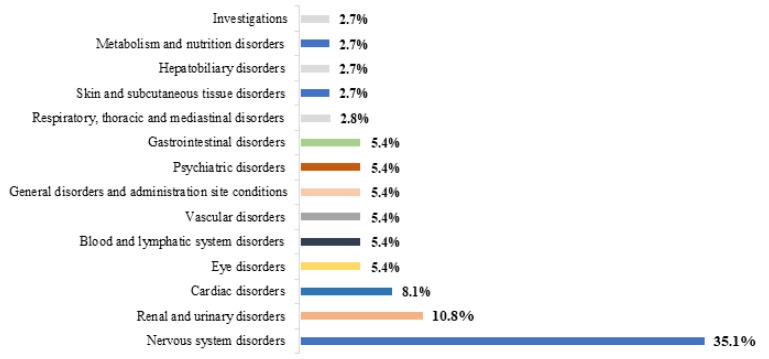
ADRs involved in DDIs in paediatric patients classified by SOCs.

Among paediatric patients, ‘nervous system’ and ‘anti-nfectives for systemic use’ were the most common ATC groups implicated in ADRs, accounting for 64.6% and 18.8%, respectively (Figure 1). ‘Nervous system disorders’ (35.1%) and ‘renal and urinary disorders’ (10.8%) were the most frequently implicated MedDRAs SOCs in associated ADRs (Figure 2).

Among these, a total of 13 serious ADRs were related to ‘contraindicated’ or ‘major’ DDIs, of which 46.2% resulted in hospitalisation.

**Figure 3:**
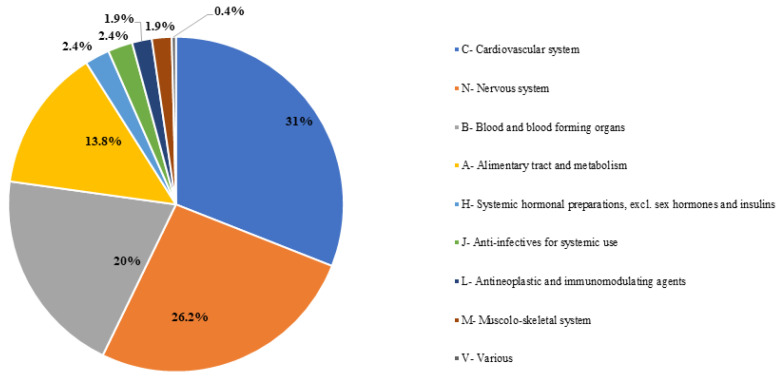
Drugs involved in DDIs in elderly patients classified by ATC code.

**Figure 4:**
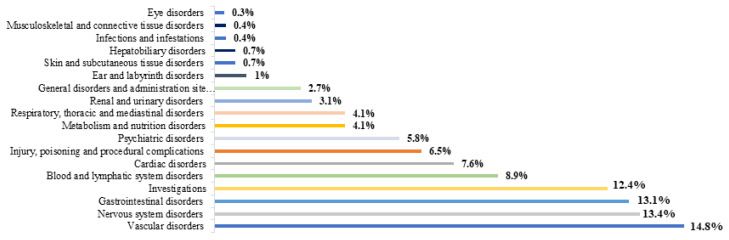
ADRs involved in DDIs in elderly patients classified by SOCs.

In reports from elderly patients, the most involved drugs in ADRs belonged to ‘cardiovascular system’ (31%), ‘nervous system’ (26.2%), and ‘blood and blood forming organs’ (20%) ATC groups (Figure 3), while ‘vascular disorders’ (14.8%), ‘nervous system disorders’ (13.4%), ‘gastrointestinal disorders’ (13.1%), and ‘investigations’ (12.4%) were the main MedDRAs SOCs implicated in related ADRs (Figure 4).

A total of 44 serious ADRs, related to ‘contraindicated’ or ‘major’ DDIs, were identified in this group of patients. Among these, 11.4% led to death, while 41% required hospitalisation.

### 3.2. Counselling

Of the 2977 reports, 95 were of pharmacological counselling, of which 49 were on paediatric patients and 46 on elderly ones. Therapeutic counselling was required for different clinical questions: 63 (66.3%) were related to the investigation of potential DDIs in complex polytherapies; 41 (43.2%) investigated drug safety and the potential occurrence of ADRs; 4 (4.2%) referred to posology and route of administration; 3 (3.2%) focused on PK issues, including questions about metabolism and specific enzyme activity or elimination that could affect safety/effectiveness profile of the therapy of interest; and 3 (3.2%) examined alternative treatments, meaning different options to cope with therapeutic management issues.

## 4. Discussion

Physiological, PK, and PD age-related changes, together with the lack of evidence on safety profile and the need for complex polytherapies, are some of the critical issues in the clinical management of paediatric and elderly patients, potentially leading to an empirical approach and unexpected DDIs and ADRs.

To our knowledge, this is the first study that provides an overview of potential DDIs and ADRs related to DDIs involving, together, paediatric and elderly patients.

The number of ADRs reported in our analysis was considerable, and the observed results were overall quite consistent with the data reported by previous studies [18].

Concerning the paediatric population, the number of reports that we analysed was higher than for the elderly one. This is in line with the evidence in the literature, according to which the prevalence of ADRs in infants and children is higher than in adults [19,20]. Our analysis did not show a difference from the gender point of view in the paediatric cases, while a prevalence of females is noticeable in the elderly group.

This latter aspect is in accordance with evidence showing a general higher susceptibility of females to ADRs occurrence due to gender differences [21,22].

One of the classes of drugs that was most frequently present in both paediatric and geriatric ADR reports belonged to the ‘anti-infectives for systemic use’ ATC group (88.6% and 68.0%, respectively). This may reflect the increased trend in the reporting rate for COVID-19 vaccines due to the pandemic and the increased awareness of people making them more prone to report ADRs. Furthermore, antibiotics were the most reported drugs in paediatric patients: this is in line with the 2021 National Report on Medicines use in Italy (OsMed), in which antimicrobials for systemic use were confirmed as the therapeutic category with the highest consumption in this group, with amoxicillin/clavulanic acid proving to be the most prescribed medicine, and azithromycin the second one, with a 19.4% increase with respect to 2020 [23]. In accordance with these data, we found that ‘anti-infectives for systemic use’ was one of the most commonly pharmacological classes implicated in DDIs and in the occurrence of related ADRs (18.8%), as supported by an 11-year analysis involving paediatric patients, performed by Bourgeois et al. [24].

Regarding potential DDIs, 251 were the ones identified in paediatric patients: 49.8% were classified as ‘moderate,’ 27.9% were ‘contraindicated,’ 20.7% were ‘major’, and only 1.6% were ‘minor’.

These results are in line with a previous study reporting 57.3% ‘moderate’ and 18% ‘major’ DDIs [25]. In contrast, another study identified 2.6% ‘contraindicated’ potential DDIs, 56.2% ‘major’, and 39.0% ‘moderate’ ones [26]. The discrepancy of these results may be ascribed to the different clinical conditions and the therapeutic regimen of paediatric inpatients [27]. Moreover, it is important to note that a portion of paediatric reports included in our analysis was related to a hospital setting: hospitalised children are more vulnerable, and they could need complex therapies, such as medications for neurological disorders and pain management [28] or require off-label use.

Concerning ADRs related to ‘contraindicated’ or ‘major’ DDIs, we identified 13 serious ADRs, of which 46.2% resulted in hospitalisation, but none of them were fatal. No fatal events were reported by Bouvy et al. either [29], who tried to justify their result by highlighting the rarity of fatal ADRs in children, or by ADRs being underreported in the studies included in the analysis.

Regarding elderly patients, 1928 potential DDIs were identified: 53% were assessed as ‘moderate’, 20% as ‘major’, 16.9% as ‘minor’, and 10.6% as ‘contraindicated’. This trend was also found by a Swedish study [30] involving over 75 patients, in which 26% of the potential DDIs were classified as ‘moderate’ and 5% as ‘major’. In accordance with our results, Juárez-Cedillo T et al. [31] identified just 2% of ‘contraindicated’ DDIs as the lowest percentage of all reports. Looking to our data, it is important to note that both elderly patients and paediatric patients showed a similar risk of developing ‘moderate’ DDIs; however, paediatric patients are more prone to develop ‘contraindicated’ DDIs with respect to the elderly population who showed a higher probability of developing ‘major’ DDIs.

The ‘cardiovascular system’ class was the most frequently pharmacological class involved in DDIs causing ADRs in geriatric patients (31%). This result may be justified by the frequency of reporting ADRs: ‘cardiovascular system’ was the most reported ATC group (89.6%) in this population, in line with previous evidence [32,33]. Indeed, cardiovascular disease is the most common cause of mortality among over-65 patients, mainly because of the presence of coronary artery disease [34]; cardiovascular drugs are indeed widely used as therapeutic classes in these patients [35,36].

Regarding ADRs related to ‘contraindicated’ or ‘major’ DDIs, a total of 44 serious ADRs were identified in this group of patients. Among these, 11.4% were fatal, whereas 41% led to hospitalisation.

The study conducted by Budnitz et al. [37] showed that patients aged 65 years or more have a higher probability of experiencing an ADR (annual estimate: 4.9 vs. 2.0 per 1000; rate ratio [RR]: 2.4; 95% confidence interval [CI]: 1.8–3.0) and requiring hospitalisation (annual estimate: 1.6 vs. 0.23 per 1000; RR: 6.8; 95%CI: 4.3–9.2) in comparison with younger individuals.

According to our results, the other most commonly implicated drugs in ADRs reporting were those referred to as the ‘alimentary tract and metabolism’ (41%) ATC groups. This could be linked to the high prevalence of the administration of hypoglycaemic drugs. Indeed, diabetes is a significant and growing public health issue, especially in older adults [38]. Moreover, it has been demonstrated that patients with diabetes receive on average four diabetes-related medications [39].

The frequent use of proton pomp inhibitors (PPIs) is another possible explanation for such a significant percentage of the ATC A group. PPIs are indeed widely used by elderly people, especially by ones who are under polytherapy [40,41]. PPIs may be a source of potentially clinically relevant DDIs due to their mechanisms of action and their influence on the absorption and metabolism of other drugs [42]. This represents an important issue since this therapeutical class is often overprescribed, even when PPIs are not necessary [43,44,45].

Polytherapy in elderly patients is very frequent, with a progressive growth in the number of different active ingredients, which increase with age. In 2021, each user took 7.4 different substances, on average, with a lower value (5.8 substances per user) recorded in the 65–69 years age group and the highest figure (8.4 substances per user) in people aged 85 and over [23].

Since population age is increasing and, as a consequence, people suffer from multiple long-term conditions, the prevalence of polypharmacy is set to rise [1]. Patients treated with polypharmacy are exposed to multiple potential DDIs, and this may cause treatment failure or the occurrence of ADRs, negatively influencing patients’ safety and increasing healthcare costs [46]. Thus, appropriate prescribing represents an increasingly necessary challenge to guarantee the efficacy and safety of therapies [47].

Furthermore, critically ill patients, including hospitalised children with complex chronic conditions, are at an even further increased risk for DDIs, not only due to the complexity of therapies but also to the physiological dysfunction arising from critical illness [48,49].

### Limitations

An important issue in this study is related to the quality of the data, which may be affected by the accuracy of the monitor. Also, the clinician’s and patients’ precision in reporting may have affected the quality of the data (i.e., the lack of information regarding the number of concomitant medications or the anamnesis of patients), and consequently the analysis. Another limitation is the lack of denominator data (number of patients prescribed the product) because neither the incidence of ADRs nor the absolute measures of risk can be estimated from our data. Furthermore, the project inception was not the same in the four regions involved. Finally, because of the nature of the study, we cannot exclude underreporting, which increased during the pandemic period.

## 5. Conclusions

The risks associated with polypharmacy are mainly due to the occurrence of DDIs, responsible for potentially clinically relevant and, sometimes, unexpected adverse events; these aspects still represent a common problem in the fragile population. In the context of therapies’ appropriateness, active pharmacovigilance studies are the best tool to allow for the identification, collection, and evaluation of complex therapies in these patients.

The MEAP 3.0 project has proved itself as a valid strategy to raise awareness of critical pharmacological issues in these vulnerable populations in order to reduce potential DDIs and the occurrence of ADRs.

The strength of this pharmacovigilance active project was the possibility of taking advantage of the professional skills of a multidisciplinary team, through the counselling tool, with the aim of selecting the appropriate therapy for each patient. Drug responsiveness is indeed characterised by individual features. The analysis of the therapy of each patient, by enhancing the appropriateness of the prescription, could be considered one of the best strategies to personalise therapy, and in doing so reduce drug-related events.

## Figures and Tables

**Table 1:** Patient characteristics.

Characteristics		Under-18 n = 1513	Over-65 n = 1464
**Age, y**			
	Mean (SD)	6.8 (5.8)	75.7 (7.6)
**Gender,** **n (%)**			
	Male	765 (50.6)	540 (36.9)
	Female	748 (49.5)	924 (63.1)
**Concomitant disease,** **n (%)**			
	Blood and lymphatic system disorders	1 (0.07)	13 (0.9)
	Cardiac disorders	1 (0.07)	47 (3.2)
	Congenital, familial, and genetic disorders	5 (0.3)	8 (0.5)
	Ear and labyrinth disorders	-	1 (0.1)
	Endocrine disorders	-	32 (2.2)
	Eye disorders	1 (0.07)	12 (0.8)
	Gastrointestinal disorders	2 (0.1)	16 (0.9)
	General disorders and administration site conditions	-	5 (0.3)
	Hepatobiliary disorders	1 (0.07)	8 (0.5)
	Immune system disorders	8 (0.5)	42 (2.9)
	Infections and infestations	4 (0.3)	18 (1.2)
	Injury, poisoning, and procedural complications	-	1 (0.1)
	Metabolism and nutrition disorders	3 (0.2)	52 (3.5)
	Musculoskeletal and connective tissue disorders	-	24 (1.6)
	Neoplasms benign, malignant, and unspecified (incl. cysts and polyps)	2 (0.1)	16 (1.1)
	Nervous system disorders	6 (0.4)	44 (3)
	Pregnancy, puerperium, and perinatal conditions	-	1 (0.1)
	Psychiatric disorders	4 (0.3)	16 (1.1)
	Renal and urinary disorders	-	19 (1.3)
	Reproductive system and breast disorders	-	1 (0.1)
	Respiratory, thoracic, and mediastinal disorders	-	17 (1.2)
	Skin and subcutaneous tissue disorders	2 (0.1)	7 (0.5)
	Social circumstances	-	3 (0.2)
	Surgical and medical procedures	-	9 (0.6)
	Vascular disorders	-	84 (5.7)
**Treatment,** **n (%)**			
	A—Alimentary tract and metabolism	193 (12.8)	600 (41)
	B—Blood and blood forming organs	23 (1.5)	478 (32.6)
	C—Cardiovascular system	31 (2.1)	1312 (89.6)
	D—Dermatologicals	17 (1.1)	16 (1.1)
	G—Genitourinary system and sex hormones	7 (0.5)	82 (5.6)
	H—Systemic hormonal preparations, excl. sex hormones and insulins	72 (4.8)	109 (7.4)
	J—Anti-infectives for systemic use	1341 (88.6)	996 (68)
	L—Antineoplastic and immunomodulating agents	126 (8.3)	217 (14.8)
	M—Muscolo-skeletal system	66 (4.4)	102 (7)
	N—Nervous system	337 (22.3)	552 (37.7)
	P—antiparasitic products, insecticides, and repellents	8 (0.5)	7 (0.5)
	R—Respiratory system	68 (4.5)	77 (5.3)
	S—Sensory organs	33 (2.2)	40 (2.7)
	V—Various	5 (0.3)	25 (1.7)

n: number of patients; y: years.

## Data Availability

Data available on request.

## Supplementary Materials

The following supporting information can be downloaded at: https://www.mdpi.com/article/10.3390/jcm12237447/s1, Figure S1: Counselling tool.

## References

[1] Integrated Health Services, Medication without Harm (2019). Medication Safety in Polypharmacy.

[2] Corsonello A., Pedone C., Incalzi R.A. (2010). Age-related pharmacokinetic and pharmacodynamic changes and related risk of adverse drug reactions. Curr. Med. Chem..

[3] Hailu B.Y., Berhe D.F., Gudina E.K., Gidey K., Getachew M. (2020). Drug related problems in admitted geriatric patients: The impact of clinical pharmacist interventions. BMC Geriatr..

[4] Esumi S., Ushio S., Zamami Y. (2022). Polypharmacy in Older Adults with Alzheimer’s Disease. Medicina.

[5] Page A.T., Potter K., Naganathan V., Hilmer S., McLachlan A.J., Lindley R.I., Coman T., Mangin D., Etherton-Beer C. (2023). Polypharmacy and medicine regimens in older adults in residential aged care. Arch. Gerontol. Geriatr..

[6] Sternberg S.A., Guy-Alfandary S., Rochon P.A. (2021). Prescribing cascades in older adults. CMAJ.

[7] Mangoni A.A., Jackson S.H. (2004). Age-related changes in pharmacokinetics and pharmacodynamics: Basic principles and practical applications. Br. J. Clin. Pharmacol..

[8] Turnheim K. (2003). When drug therapy gets old: Pharmacokinetics and pharmacodynamics in the elderly. Exp. Gerontol..

[9] Joseph P.D., Craig J.C., Caldwell P.H. (2015). Clinical trials in children. Br. J. Clin. Pharmacol..

[10] O’Hara K. (2016). Paediatric pharmacokinetics and drug doses. Aust. Prescr..

[11] Carnovale C., Brusadelli T., Zuccotti G., Beretta S., Sullo M.G., Capuano A., Rossi F., Moschini M., Mugelli A., Vannacci A. (2014). The importance of monitoring adverse drug reactions in pediatric patients: The results of a national surveillance program in Italy. Expert Opin. Drug Saf..

[12] Van Spall H.G., Toren A., Kiss A., Fowler R.A. (2007). Eligibility criteria of randomized controlled trials published in high-impact general medical journals: A systematic sampling review. JAMA.

[13] Meng M., Zhou Q., Lei W., Tian M., Wang P., Liu Y., Sun Y., Chen Y., Li Q. (2022). Recommendations on Off-Label Drug Use in Pediatric Guidelines. Front. Pharmacol..

[14] INTERCheckWEB. https://intercheckweb.marionegri.it/.

[15] ICH E2A Clinical Safety Data Management: Definitions and Standards for Expedited Reporting—Scientific Guideline. https://www.ema.europa.eu/en/ich-e2a-clinical-safety-data-management-definitions-standards-expedited-reporting-scientific#current-effective-version-section.

[16] MedDRA Web-Based Browser. https://tools.meddra.org/wbb/.

[17] ATC/DDD Index. https://www.whocc.no/atc_ddd_index/.

[18] Khan Z., Karataş Y., Kıroğlu O. (2021). Evaluation of Adverse Drug Reactions in Paediatric Patients: A Retrospective Study in Turkish Hospital. Front. Pharmacol..

[19] Leporini C., De Sarro C., Palleria C., Caccavo I., Piro B., Citraro R., De Sarro G. (2022). Pediatric Drug Safety Surveillance: A 10-Year Analysis of Adverse Drug Reaction Reporting Data in Calabria, Southern Italy. Drug Saf..

[20] Nasso C., Mecchio A., Rottura M., Valenzise M., Menniti-Ippolito F., Cutroneo P.M., Squadrito V., Squadrito F., Pallio G., Irrera N. (2020). A 7-Years Active Pharmacovigilance Study of Adverse Drug Reactions Causing Children Admission to a Pediatric Emergency Department in Sicily. Front. Pharmacol..

[21] Hendriksen L.C., van der Linden P.D., Lagro-Janssen A.L.M., van den Bemt P.M.L.A., Siiskonen S.J., Teichert M., Kuiper J.G., Herings R.M.C., Stricker B.H., Visser L.E. (2021). Sex differences associated with adverse drug reactions resulting in hospital admissions. Biol. Sex Differ..

[22] Haile D.B., Ayen W.Y., Tiwari P. (2013). Prevalence and assessment of factors contributing to adverse drug reactions in wards of a tertiary care hospital, India. Ethiop. J. Health Sci..

[23] Italian Medicines Agency, The Medicines Utilisation Monitoring Centre (2022). National Report on Medicines Use in Italy—Year 2021.

[24] Bourgeois F.T., Mandl K.D., Valim C., Shannon M.W. (2009). Pediatric adverse drug events in the outpatient setting: An 11-year national analysis. Pediatrics.

[25] Hassanzad M., Arenas-Lopez S., Baniasadi S. (2018). Potential Drug-Drug Interactions Among Critically Ill Pediatric Patients in a Tertiary Pulmonary Center. J. Clin. Pharmacol..

[26] Choi Y.H., Lee I.H., Yang M., Cho Y.S., Jo Y.H., Bae H.J., Kim Y.S., Park J.D. (2021). Clinical significance of potential drug-drug interactions in a pediatric intensive care unit: A single-center retrospective study. PLoS ONE.

[27] Bebitoğlu B.T., Oğuz E., Nuhoğlu Ç., Dalkılıç A.E.K., Çirtlik P., Temel F., Hodzic A. (2020). Evaluation of potential drug-drug interactions in a pediatric population. Turk Pediatr. Ars..

[28] O’Donnell F.T., Rosen K.R. (2014). Pediatric pain management: A review. Mo. Med..

[29] Bouvy J.C., De Bruin M.L., Koopmanschap M.A. (2015). Epidemiology of adverse drug reactions in Europe: A review of recent observational studies. Drug Saf..

[30] Johnell K., Klarin I. (2007). The relationship between number of drugs and potential drug-drug interactions in the elderly: A study of over 600,000 elderly patients from the Swedish Prescribed Drug Register. Drug Saf..

[31] Juárez-Cedillo T., Martinez-Hernández C., Hernández-Constantino A., Garcia-Cruz J.C., Avalos-Mejia A.M., Sánchez-Hurtado L.A., Islas Perez V., Hansten P.D. (2016). Clinical Weighting of Drug-Drug Interactions in Hospitalized Elderly. Basic Clin. Pharmacol. Toxicol..

[32] Jiang H., Lin Y., Ren W., Fang Z., Liu Y., Tan X., Lv X., Zhang N. (2022). Adverse drug reactions and correlations with drug-drug interactions: A retrospective study of reports from 2011 to 2020. Front. Pharmacol..

[33] Howard R.L., Avery A.J., Slavenburg S., Royal S., Pipe G., Lucassen P., Pirmohamed M. (2007). Which drugs cause preventable admissions to hospital? A systematic review. Br. J. Clin. Pharmacol..

[34] Duncan A.K., Vittone J., Fleming K.C., Smith H.C. (1996). Cardiovascular disease in elderly patients. Mayo Clin. Proc..

[35] Schwartz J.B., Schmader K.E., Hanlon J.T., Abernethy D.R., Gray S., Dunbar-Jacob J., Holmes H.M., Murray M.D., Roberts R., Joyner M. (2019). Pharmacotherapy in Older Adults with Cardiovascular Disease: Report from an American College of Cardiology, American Geriatrics Society, and National Institute on Aging Workshop. J. Am. Geriatr. Soc..

[36] Mohsenzadeh P., Ardekani A., Poustchi H., Mohammadi Z., Abdipour Mehrian S.R., Bazrafshan Drissi H., Rahimian Z., Taherifard E., Nabavizadeh A., Kamalipour A. (2022). Population-based pattern of medication use and prevalence of polypharmacy among patients with cardiovascular diseases: Results of the Pars cohort study from Iran. BMC Cardiovasc. Disord..

[37] Budnitz D.S., Pollock D.A., Weidenbach K.N., Mendelsohn A.B., Schroeder T.J., Annest J.L. (2006). National surveillance of emergency department visits for outpatient adverse drug events. J. Am. Med. Assoc..

[38] Cowie C.C., Casagrande S.S., Menke A., Cissell M.A., Eberhardt M.S., Meigs J.B., Gregg E.W., Knowler W.C., Barrett-Connor E., Becker D.J. (2018). Diabetes in America.

[39] Huang E.S., Basu A., Finch M., Frytak J., Manning W. (2007). The complexity of medication regimens and test ordering for patients with diabetes from 1995 to 2003. Curr. Med. Res. Opin..

[40] Jaynes M., Kumar A.B. (2008). The risks of long-term use of proton pump inhibitors: A critical review. Ther. Adv. Drug Saf..

[41] Nardino R.J., Vender R.J., Herbert P.N. (2000). Overuse of acid-suppressive therapy in hospitalized patients. Am. J. Gastroenterol..

[42] Wedemeyer R.S., Blume H. (2014). Pharmacokinetic drug interaction profiles of proton pump inhibitors: An update. Drug Saf..

[43] Pasina L., Nobili A., Tettamanti M., Salerno F., Corrao S., Marengoni A., Iorio A., Marcucci M., Mannucci P.M. (2011). Prevalence and Appropriateness of Drug Prescriptions for Peptic Ulcer and Gastro-Esophageal Reflux Disease in a Cohort of Hospitalized Elderly. Eur. J. Int. Med..

[44] Galai E., Scotti L., Gilardetti M., Ucciero A., Ferrante D., Poluzzi E., Genazzani A.A., Barone-Adesi F. (2022). Time-Trends of Drug-Drug Interactions among Elderly Outpatients in the Piedmont Region (Italy): A Population-Based Study. Int. J. Environ. Res. Public Health.

[45] Cena C., Traina S., Parola B., Bo M., Fagiano R., Siviero C. (2020). Prescription of proton pump inhibitors in older adults with complex polytherapy. Eur. J. Hosp. Pharm..

[46] Dai D., Feinstein J.A., Morrison W., Zuppa A.F., Feudtner C. (2016). Epidemiology of Polypharmacy and Potential Drug-Drug Interactions Among Pediatric Patients in ICUs of U.S. Children’s Hospitals. Pediatr. Crit. Care Med..

[47] Mallet L., Spinewine A., Huang A. (2007). The challenge of managing drug interactions in elderly people. Lancet.

[48] Mouly S., Meune C., Bergmann J.F. (2009). Mini-series: I. Basic science. Uncertainty and inaccuracy of predicting CYP-mediated in vivo drug interactions in the ICU from vitro models: Focus on CYP3A4. Intensive Care Med..

[49] Askari M., Eslami S., Louws M., Wierenga P.C., Dongelmans D.A., Kuiper R.A., Abu-Hanna A. (2013). Frequency and nature of drug-drug interactions in the intensive care unit. Pharmacoepidemiol. Drug Saf..

