# Supplementary material for: Two Years of Active Pharmacovigilance Surveillance and Therapeutic Reconciliation in Frail Populations: The MEAP 3.0 Study
Source: J Clin Med. 2023 Nov 30;12(23):7447. doi: 10.3390/jcm12237447 (PMC10707211; doi:10.3390/jcm12237447)
Supplement: Supplementary file 1 [file jcm-12-07447-s001.zip › jcm-2718129-supplementary.pdf]

Supplementary Figure S1. Counselling tool.

COUNSELLING TOOL

Patient's first and last name ______________ _____Sex ____________________________________

Date of birth______________________ Birthplace________ _____________________________

Weight___________Unit__________________________________Bed_____________________

Admission Date ____

THERAPY

RECOGNITION			RECONCILIATION				
Therapy	Dose	Route of administration	Therapy withdrawal	Change in therapy	No therapy interruption	New therapy	Comments

Clinical question

Pharmacological analysis
